# Supplementary material for: Diagnostic accuracy of midkine on hepatocellular carcinoma: A meta-analysis
Source: PLoS One. 2019 Oct 10;14(10):e0223514. doi: 10.1371/journal.pone.0223514 (PMC6786585; doi:10.1371/journal.pone.0223514)
Supplement: S1 Fig — (DOCX) [file pone.0223514.s001.docx]

PubMed:

#1 liver [MeSH Terms]

#2 liver

#3 hepatocellular

#4 hepatic

#5 OR/1-4

#6 neoplasms [MeSH Terms]

#7 neoplasms

#8 neoplasm

#9 carcinoma [MeSH Terms]

#10 carcinoma

#11 tumour

#12 tumor

#13 cancer

#14 OR/6-13

#15 midkine [MeSH Terms]

#16 midkine

#17 mdk

#18 OR/15-17

#19 sensitivity and specificity [MeSH Terms]

#20 sensitivity

#21 specificity

#22 OR/19-21

#23 roc curve [MeSH Terms]

#24 roc curve

#25 roc

#26 curve

#27 AND/25-26

#28 OR/23-24, 27

#29 diagnosis [MeSH Terms]

#30 diagnosis

#31 diagnostic

#32 OR/29-31

#33 OR/22, 28, 32

#34 AND/5, 14, 18, 33

Embase:

#1 'liver'/exp

#2 liver

#3 hepatocellular

#4 hepatic

#5 OR/1-4

#6 'carcinoma'/exp

#7 carcinoma

#8 'neoplasm'/exp

#9 neoplasm

#10 'tumour'/exp

#11 tumour

#12 'tumor'/exp

#13 tumor

#14 'cancer'/exp

#15 cancer

#16 OR/6-15

#17 'midkine'/exp

#18 midkine

#19 mdk

#20 OR/17-19

#21 'sensitivity'/exp

#22 sensitivity

#23 'specificity'/exp

#24 specificity

#25 'roc curve'/exp

#26 roc curve

#27 roc

#28 'curve'/exp

#29 curve

#30 OR/28-29

#31 AND/27, 30

#32 'diagnosis'/exp

#33 diagnosis

#34 'diagnostic'/exp

#35 diagnostic

#36 OR/21-26, 31-35

#37 AND/5, 16, 20, 36
