# Supplementary material for: Diagnostic accuracy of midkine on hepatocellular carcinoma: A meta-analysis
Source: PLoS One. 2019 Oct 10;14(10):e0223514. doi: 10.1371/journal.pone.0223514 (PMC6786585; doi:10.1371/journal.pone.0223514)
Supplement: S2 Table — (DOCX) [file pone.0223514.s003.docx]

**S2 Table**. Diagnostic accuracy of the included studies

| Author | Year | TP | FP | FN | TN | Sensitivity (95% CI) | Specificity (95% CI) |
| --- | --- | --- | --- | --- | --- | --- | --- |
| Habachi *et al* | 2018 | 86 | 9 | 0 | 80 | 1.00 (0.96-1.00) | 0.90 (0.82-0.95) |
| Hodeib *et al* | 2017 | 32 | 0 | 3 | 35 | 0.91 (0.78-0.97) | 1.00 (0.90-1.00) |
| Hung *et al* | 2011 | 43 | 24 | 29 | 96 | 0.60 (0.48-0.70) | 0.80 (0.72-0.86) |
| Li *et al* | 2006 | 87 | 8 | 17 | 52 | 0.84 (0.75-0.90) | 0.87 (0.76-0.93) |
| Mashaly *et al* | 2018 | 36 | 5 | 8 | 26 | 0.82 (0.68-0.91) | 0.84 (0.67-0.93) |
| Saad *et al* | 2013 | 26 | 16 | 3 | 29 | 0.90 (0.74-0.96) | 0.64 (0.50-0.77) |
| Shaheen *et al* | 2015 | 37 | 5 | 3 | 25 | 0.93 (0.80-0.97) | 0.83 (0.66-0.93) |
| Shaheen *et al* | 2015 | 40 | 1 | 0 | 29 | 1.00 (0.91-1.00) | 0.97 (0.83-0.99) |
| Vongsuvanh *et al* | 2016 | 61 | 65 | 25 | 107 | 0.71 (0.61-0.80) | 0.62 (0.55-0.69) |
| Zhu *et al* | 2013 | 219 | 81 | 33 | 374 | 0.87 (0.82-0.91) | 0.82 (0.78-0.85) |
| Zhu *et al* | 2013 | 74 | 4 | 12 | 36 | 0.86 (0.77-0.92) | 0.90 (0.77-0.96) |

Abbreviations: TP true positive, FP false positive, FN false negative, TN true negative.
