# Supplementary material for: Diagnostic accuracy of midkine on hepatocellular carcinoma: A meta-analysis
Source: PLoS One. 2019 Oct 10;14(10):e0223514. doi: 10.1371/journal.pone.0223514 (PMC6786585; doi:10.1371/journal.pone.0223514)
Supplement: S3 Table — (DOCX) [file pone.0223514.s004.docx]

| STUDY | RISK OF BIAS | | | |  | APPLICABILITY CONCERNS | | |
| --- | --- | --- | --- | --- | --- | --- | --- | --- |
|  | PATIENT SELECTION | INDEX  TEST | REFERENCE STANDARD | FLOW AND TIMING |  | PATIENT SELECTION | INDEX  TEST | REFERENCE STANDARD |
| Habachi *et al*,  2018 | ☹ | ? | ☹ | ☹ |  | ☺ | ☺ | ? |
| Hodeib *et al*,  2017 | ☹ | ☹ | ☺ | ☺ |  | ☺ | ☺ | ☺ |
| Hung *et al*,  2011 | ? | ? | ☹ | ? |  | ☺ | ☺ | ? |
| Li *et al*,  2006 | ☹ | ☹ | ☺ | ? |  | ☺ | ☺ | ☺ |
| Mashaly *et al*,  2018 | ? | ? | ☺ | ☺ |  | ☺ | ☺ | ☺ |
| Saad *et al*,  2013 | ☹ | ? | ☺ | ? |  | ☺ | ☺ | ☺ |
| Shaheen *et al*,  2015 | ? | ? | ☺ | ☺ |  | ☺ | ☺ | ☺ |
| Vongsuvanh *et al*,  2016 | ☹ | ? | ☺ | ☺ |  | ☺ | ☺ | ☺ |
| Zhu *et al*,  2013 | ☺ | ☺ | ☺ | ☺ |  | ☺ | ☺ | ☺ |

**S3 Table**. Quality assessment for 9 studies using QUADAS-2

☺Low Risk ☹High Risk ?Unclear Risk
