# Supplementary material for: Diagnostic accuracy of midkine on hepatocellular carcinoma: A meta-analysis
Source: PLoS One. 2019 Oct 10;14(10):e0223514. doi: 10.1371/journal.pone.0223514 (PMC6786585; doi:10.1371/journal.pone.0223514)
Supplement: Attachment — Submitted filename: Response to Reviewers.docx [file pone.0223514.s005.docx]

**Response to Reviewers’ comments**

**Reviewer #1 (Remarks to the Author):**

Dr. Zhang and colleagues made a comprehensive review of literature on accuracy of midkine for the diagnosis of HCC.

However,

- Remove “holistic” from introduction section

**Response:** Thanks for reviewer’s comments. We have removed “holistic” from introduction section.

- In the result section, authors decribe the control group without HCC as composed by 113 patients with chronic hepatitis C or HBV, without comments on possible DAA or NUC treatment, and by 453 subjects with liver cirrhosis, without specify the etiology of liver disease. In discussion section, this limit should be pointed out and highlighted, as well as the AFP levels used (20-200 ng/mL) for the accuracy comparison are very hight. According to literature, accuracy of AFP for HCC changes according to etiology, and nowadays lower AFP thresholds are under evaluation for increasing HCC surveillance.

**Response:** The reviewer’s suggestion is highly appreciated. As suggested, we have highlighted these limits in the discussion section to conclude our results more precisely.

- Add recent EASL HCC guidelines in references section and introduction

**Response:** Thanks for reviewer's comments. As suggested, we have added recent EASL HCC guidelines (EASL Clinical Practice Guidelines: Management of hepatocellular carcinoma, doi: 10.1016/j.jhep.2018.03.019) in references section and introduction.

- Only 6 studies evaluated the role of AFP vs MK, and only three AFP+MK: this point of limit numbers of studies on AFP, and moreover with different cut-off on etiology should be stressed in discussion, and also in results section of the abstract. In fact, here there are no data on AFP and MK comparison in result section, but AFP appears in the conclusion statement. Modify results section or change conclusion statement, removing AFP.

**Response:** We thank the reviewer for the valuable concerns. We have stressed in the discussion section that lack of AFP studies in the included literature and selection of different cut-off values of AFP may undermine the stability of our results. We also removed “AFP” in conclusions section of the abstract.

**Reviewer #2 (Remarks to the Author):**

This study investigated about the dependability and accuracy of midkine (MK) to diagnose HCC by systematic review and meta- analysis. In this study, 9 articles were incorporated and the authors reported that the sensitivity and specificity of MK for diagnosing HCC were 0.85 and 0.93, respectively. And furthermore, the authors indicate that the best efficient cut-off value of MK level to diagnose HCC was 0.5ng/mL. Although not entirely novel, this study is potentially interesting to the readers as the authors showed the possibility of MK as a novel diagnostic biomarker of HCC. The study design is well organized and the statistics seem to have been conducted appropriately.

However, I have several points as indicated below need to be addressed in the manuscript by the authors to improve the quality of this article.

Major points

1. According to the past report by Hung et al, serum MK levels could have potential to be useful to monitor HCC progression. Have you analyzed about the correlation between the HCC stage or prognosis of the patients with HCC and serum MK levels? Of course, I know that this research focused only about the diagnostic usefulness of MK. But if the authors have some informative data about the possibility of MK for prediction of prognosis or monitoring the response to therapy, it would be more useful to include the information about this point.

**Response:** We thank the reviewer for the predictive ability concerns about MK in HCC prognosis. To the best of our knowledge, there have been no studies on the relationship between serum MK expression level and liver cancer prognosis. But we are preparing to conduct a prospective study to investigate the association between pre- and postoperative level of serum MK and HCC prognosis.

2. Is there any difference in MK level according to the liver fibrosis status? Is the cut-off level to diagnose of HCC the same between the cirrhosis and non-cirrhosis patients? Whether the authors have some data or not, it would be useful to mention about this point in result or discussion part.

**Response:** Thanks for reviewer's constructive suggestions. After carefully reviewing all the included articles, we found that none of them mentioned the relationship between different levels of liver fibrosis and MK expression values. And there was no mention of the difference in cutoff values used by MK in diagnosing HCC patients with and without liver cirrhosis. The points were mentioned in the discussion part.

Minor point

1. “heparin” in the last line of page 20 is misspelled.

**Response:** Thank the review for pointing out this typo. As suggested, “heparan” was replaced by “heparin”.

**Reviewer #3 (Remarks to the Author):**

Please also include timeline of the literature search in the method section of the abstract.

**Response:** Thanks for the helpful comments. As suggested, we added the timeline of the literature search in the method section of the abstract.

Please also include timeline of the literature search in the method section of the Fulltext.

**Response:** Thanks for the helpful comments. As suggested, we added the timeline of the literature search in the method section of the full text.

When Pubmed is used for the search, MESH terms are always recommended to be included.

**Response:** Thanks for reviewer's suggestions. We added MESH terms in our search syntax.

Search terms in PubMed and Embase are different. Please attach syntax used in each database as supplementary.

**Response:** The reviewer’s suggestion is highly appreciated. We attached the syntax used in PubMed and Embase as supplementary.

It will be better to show kappa for the selection and data extraction. Please show the data of kappa of agreement during the systematic searches. How disagreements were solved during the systematic search among two independent reviewers?

**Response:** Thanks for reviewer's comments. Diagnostic meta-analysis comprises four key elements, including TP, FP, TN, and FN. They are different from those indexes requiring subjective calculation or evaluation, they are objective data described directly in the original text, so we did not calculate the kappa value. Disagreement was resolved through discussion or consultation with the third investigator (J-Y.Y.).

Figure1, suggest to use PRISMA 2009 Flow Diagram platform

**Response:** Thanks for reviewer's suggestion. We changed our flow diagram into PRISMA 2009 style.

Please make the data for this review publicly available, possibly through the Open Science Framework (osf.io). Items to include: list of excluded studies, commands for statistical analysis, spreadsheets or data used for the meta-analyses, etc. Making data publicly available will promote the reproducibility of the review and is best practices for systematic reviews and meta-analyses.

**Response:** Thanks for reviewer's constructive suggestions. We made our data publicly available through Open Science Framework.

Some revision of the English language is needed. There are some parts of the paper where it is quite difficult to make sense of some sentences. English edit will help to improve the quality of the manuscript.

“makes it readily to quantitate in blood samples” is not correct in grammar.

“above fifty percent HCC patients” is not correct

“Studies that with insufficient data” is not correct

“other type” is not correct in grammar.

“10 review and meta-analysis were excluded based upon” is not correct in grammar.

“literatures” is not correct in grammar. It is uncountable noun

“Jing et al concludes that MK demonstrates great performance in the diagnosis of malignant diseases like esophageal squamous cell carcinoma” is not correct. It should be past tense.

“Hung et al concludes that the longitudinal monitoring of serum MK is incapable of detecting HCC recurrence and de novo HCC” is not correct. It should be past tense.

“A recent meta-analysis concludes that US plus AFP may serve as an updated screening strategy for early HCC.” is not correct. It should be past tense.

It is not professional to use “And” at the beginning of sentences in academic writing.

**Response:** I sincerely appreciate the reviewer's serious comments. These mistakes were carefully corrected.
