# Supplementary material for: Diagnostic accuracy of midkine on hepatocellular carcinoma: A meta-analysis
Source: PLoS One. 2019 Oct 10;14(10):e0223514. doi: 10.1371/journal.pone.0223514 (PMC6786585; doi:10.1371/journal.pone.0223514)
Supplement: Attachment — Submitted filename: Response to Reviewers.docx [file pone.0223514.s006.docx]

**Response to Reviewers’ comments**

**Reviewer #2 (Remarks to the Author):**

Some comments this reviewer pointed out were properly addressed. This reviewer thought the manuscript should be accepted for publication.

**Response:** Thanks to the reviewer for his recognition and support of our paper.

**Reviewer #3 (Remarks to the Author):**

Reviewer #3: The authors claimed they included "MESH terms in our search syntax." However, this needs to be mentioned formally in the method section of manuscript. MESH term for Pubmed should be provided in the full manuscript. The authors claimed "We attached the syntax used in PubMed and Embase as supplementary." However, this is not good enough. Attached file S1 Figure.esp is not professional. This needs to be more formal in the manuscript for as .doc that easy to assess.

**Response:** We thank the reviewer for the valuable concerns. We included the “MeSH Terms” in the method section of the manuscript. And we changed “S1 Figure.esp” into “S1 Figure.docx”, which encompasses the panorama of the whole search terms. We sincerely hope that these changes will get your approval.

The authors claimed they made "data publicly available through Open Science Framework"; however, they have not provided the formal like that can access data publicly in the manuscript. Need to include in method section or the first part of result.

**Response:** The reviewer’s suggestion is highly appreciated. We have made our data publicly available through Open Science Framework, global researchers can freely access the data through the formal link ([osf.io/gw8em/](https://osf.io/gw8em/)) provided in the method part of our manuscript.

The authors claimed they made changes in English edits as "These mistakes were carefully corrected." However, there are very minor changes in the manuscript and still very difficult to read through in English writing. English language Edit is needed including formal proof.

**Response:** We thank the reviewer for language concerns. We sincerely apologize for the unsatisfactory amendments. We have sent this manuscript to American Journal Experts for professional language polishing, and the corresponding formal proof has been attached in supplement. We hope these changes may get your approval.
